# Retraction Note: Anlotinib combined with temozolomide suppresses glioblastoma growth via mediation of JAK2/STAT3 signaling pathway

**DOI:** 10.1007/s00280-026-04874-0

**Published:** 2026-03-16

**Authors:** Peng Xu, Handong Wang, Hao Pan, Jiakai Chen, Chulei Deng

**Affiliations:** 1 Department of Neurosurgery, Jinling Hospital, School of Medicine, Southeast University, 305 East Zhongshan Road, Nanjing, 210002 Jiangsu China; 2 Department of Neurosurgery, Jinling Hospital, Medical School of Nanjing University, Nanjing, 210002 Jiangsu China; 3 Department of Neurosurgery, Jinling Hospital, The First School of Clinical Medicine, Southern Medical University, Nanjing, 210002 China

**Retraction Note:**

**Cancer Chemotherapy and Pharmacology (2022) 89:183–196**

10.1007/s00280-021-04380-5

The Editors-in-Chief have retracted this article.

Following publication, the authors requested a correction to Figure 2 due to overlaps in the raw images. During further checks, the Publisher identified image overlaps in Figure 4 and a mismatch between the ethics approval committee name in the published article and the submitted document.

The Editors-in-Chief therefore, have lost confidence in the results and conclusions of this article.

The authors did not respond to correspondence from the Publisher about this retraction.

Concerns:

- Fig. 2C Migration, A172, 0 µM appears to overlap with A251, 0 µM;
- Fig. 2E, Invasion, U87 0 µM appears to overlap with U251 0 µM;
- Fig. 4G p-STAT3, A172 appears to overlap with Fig. 5F p-STAT3, U251

